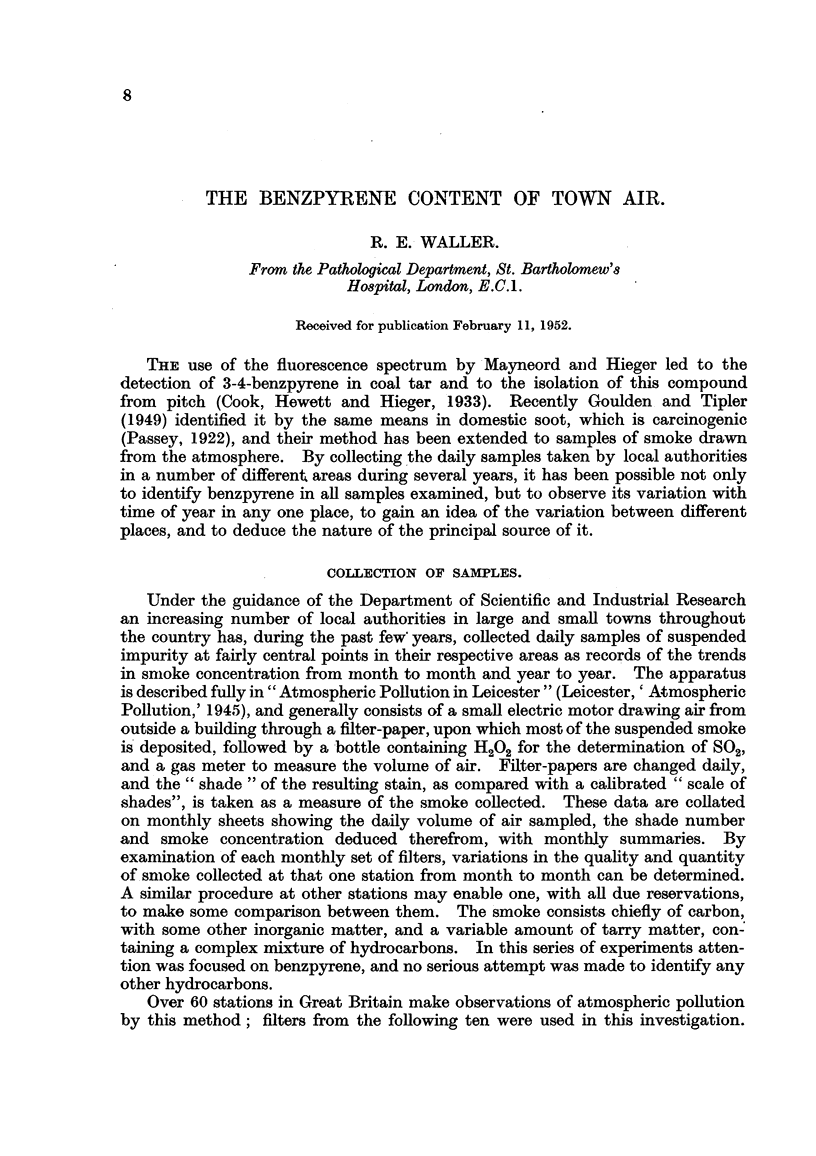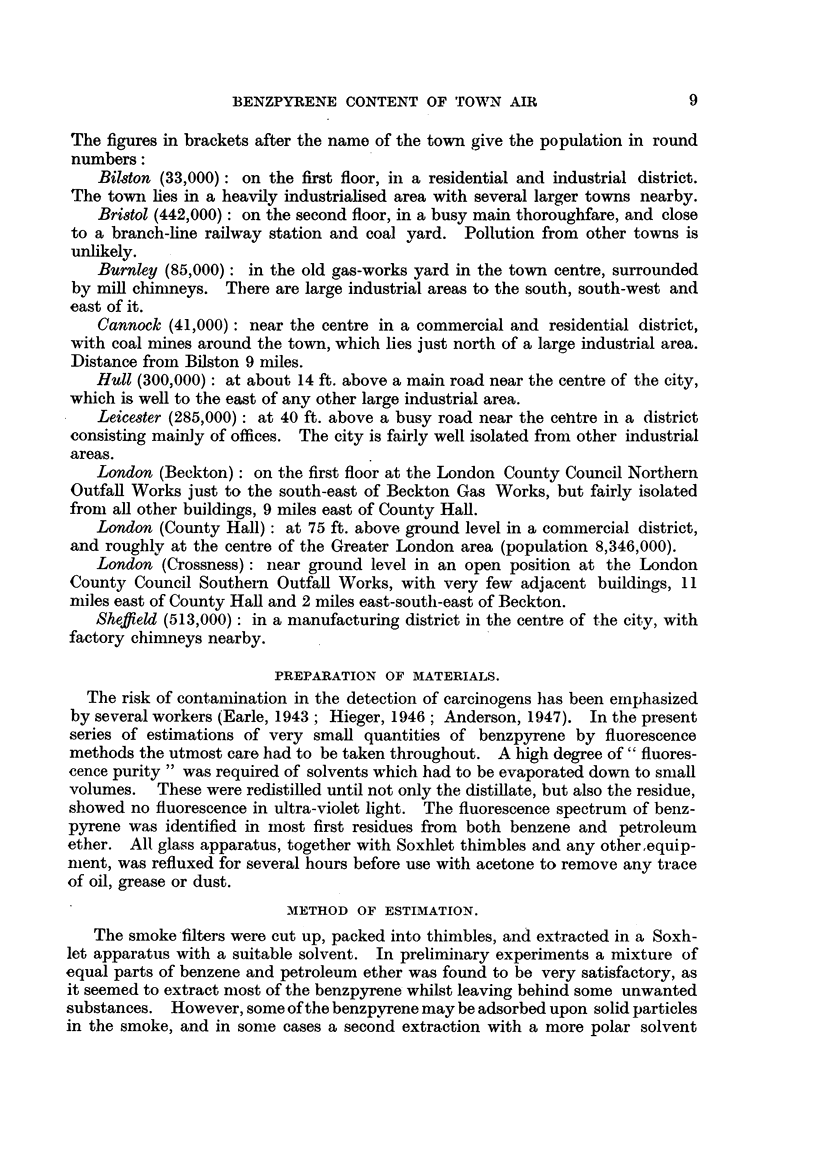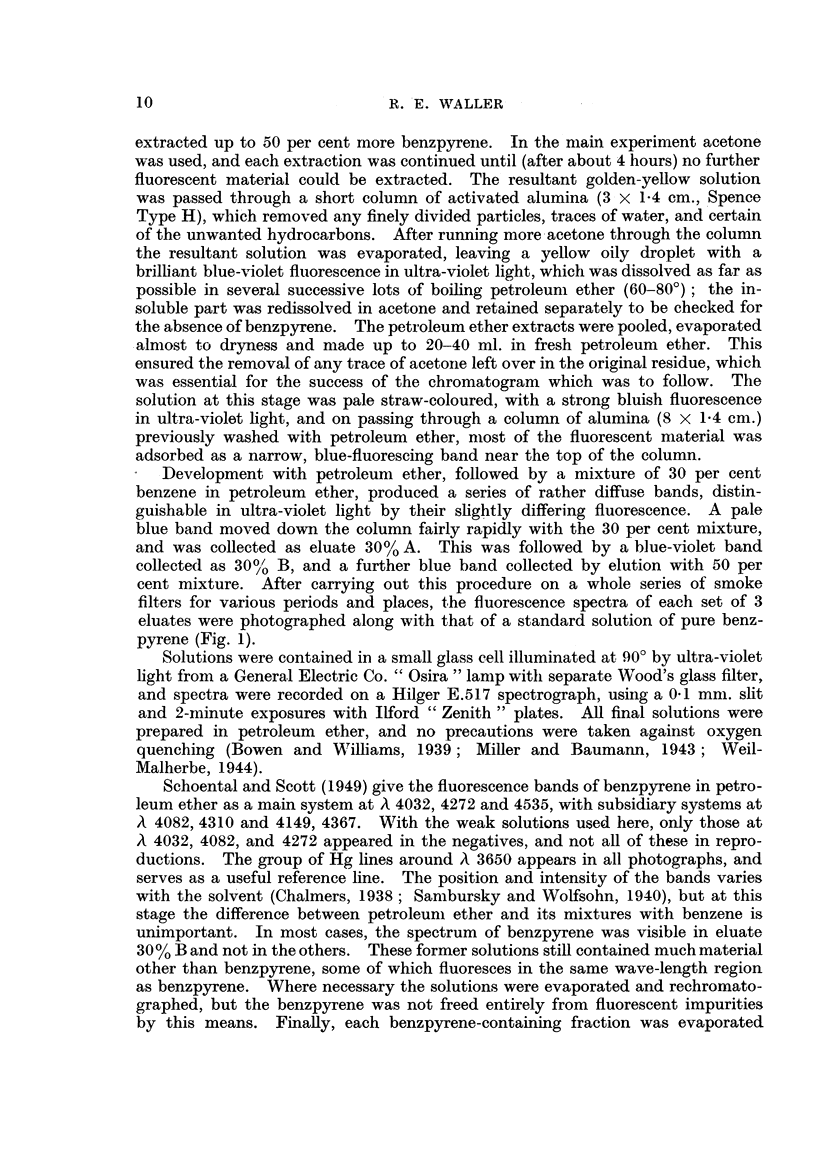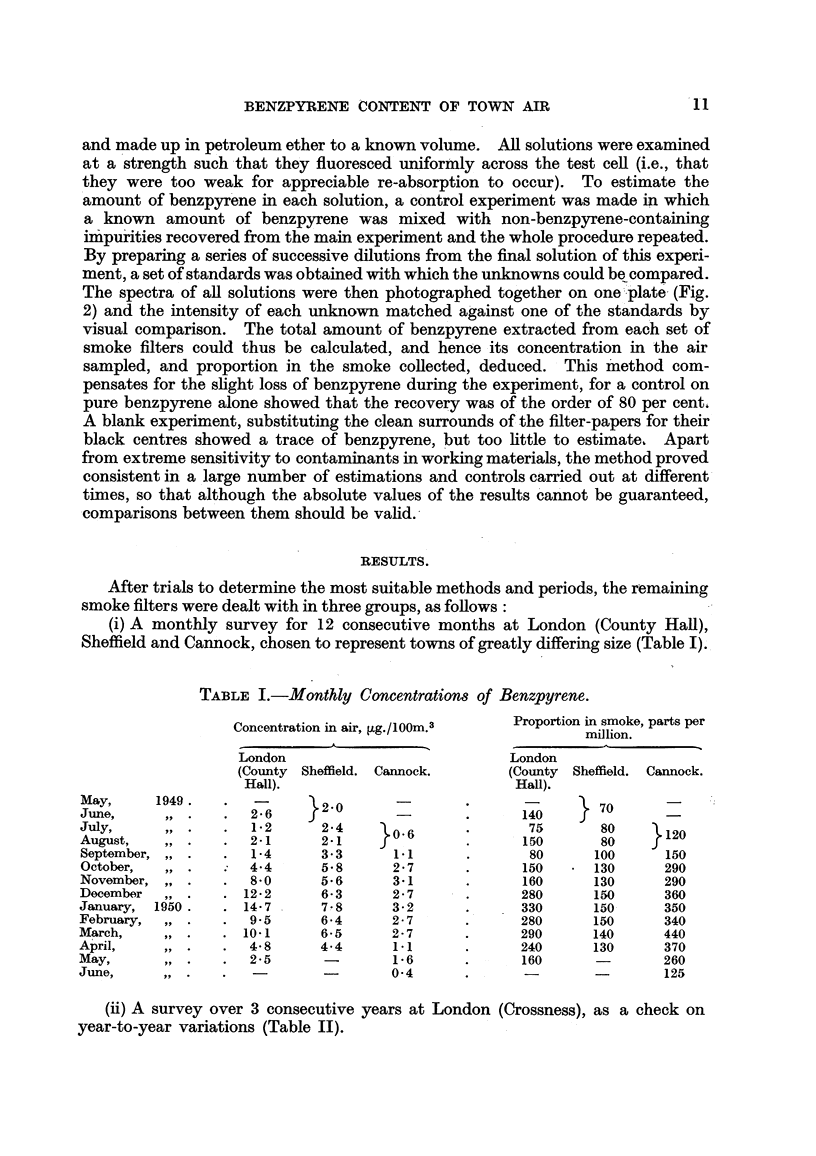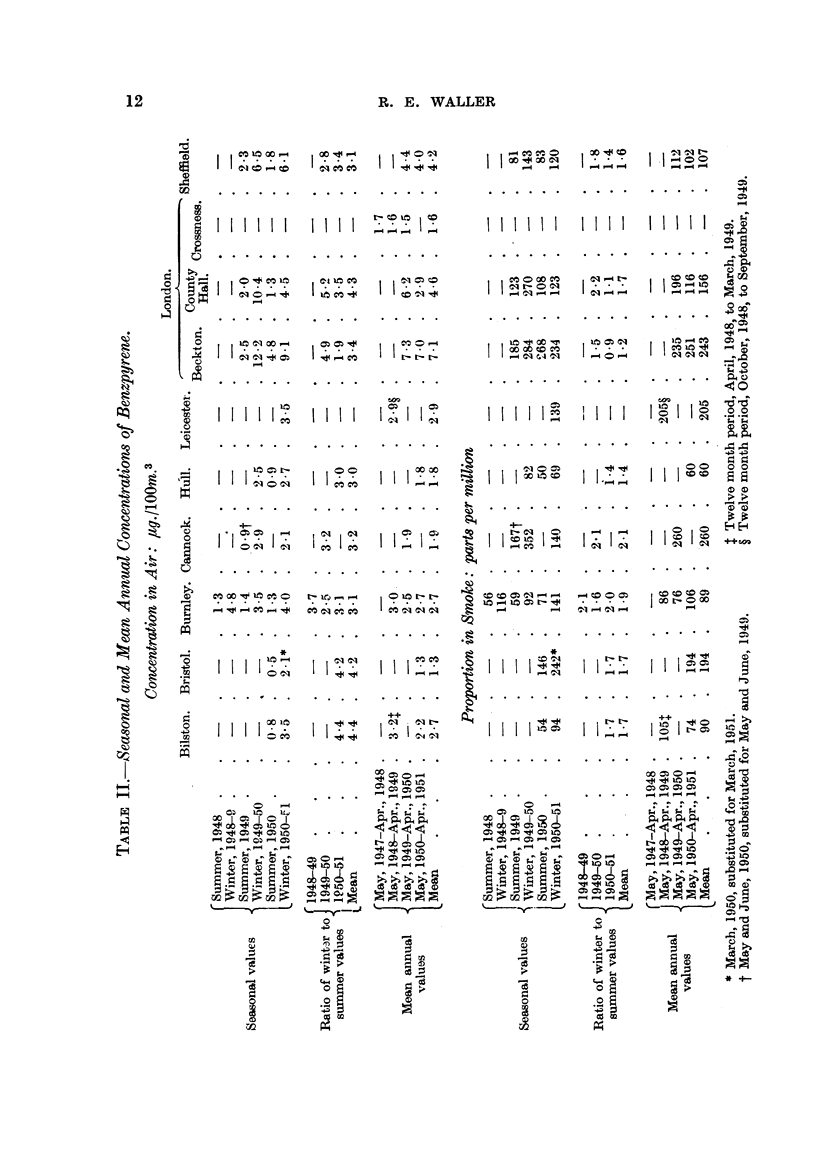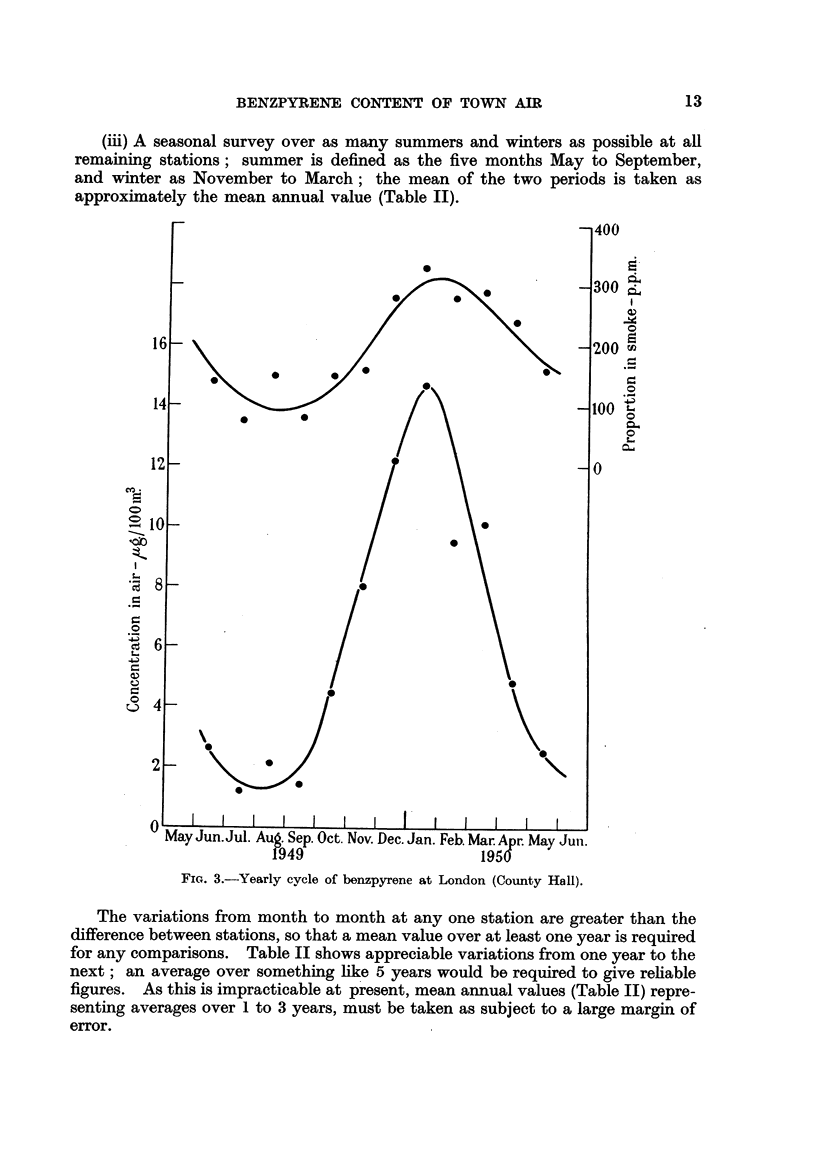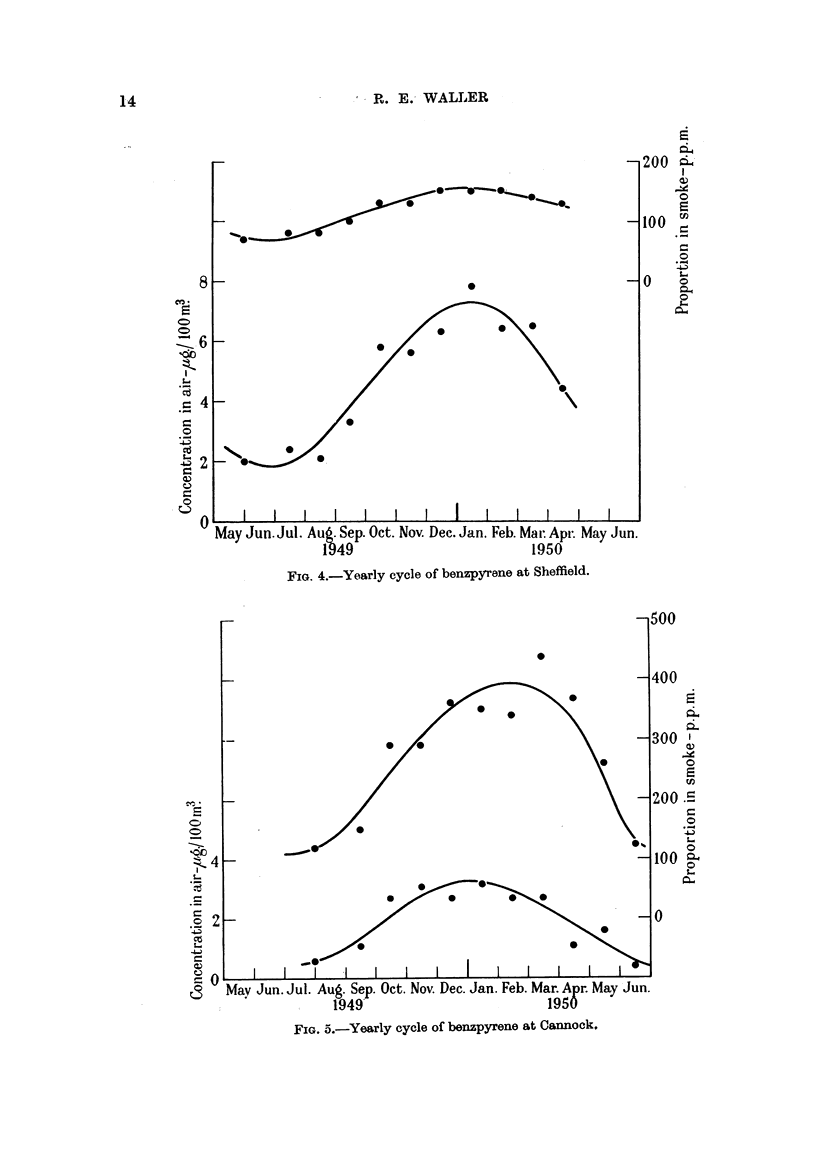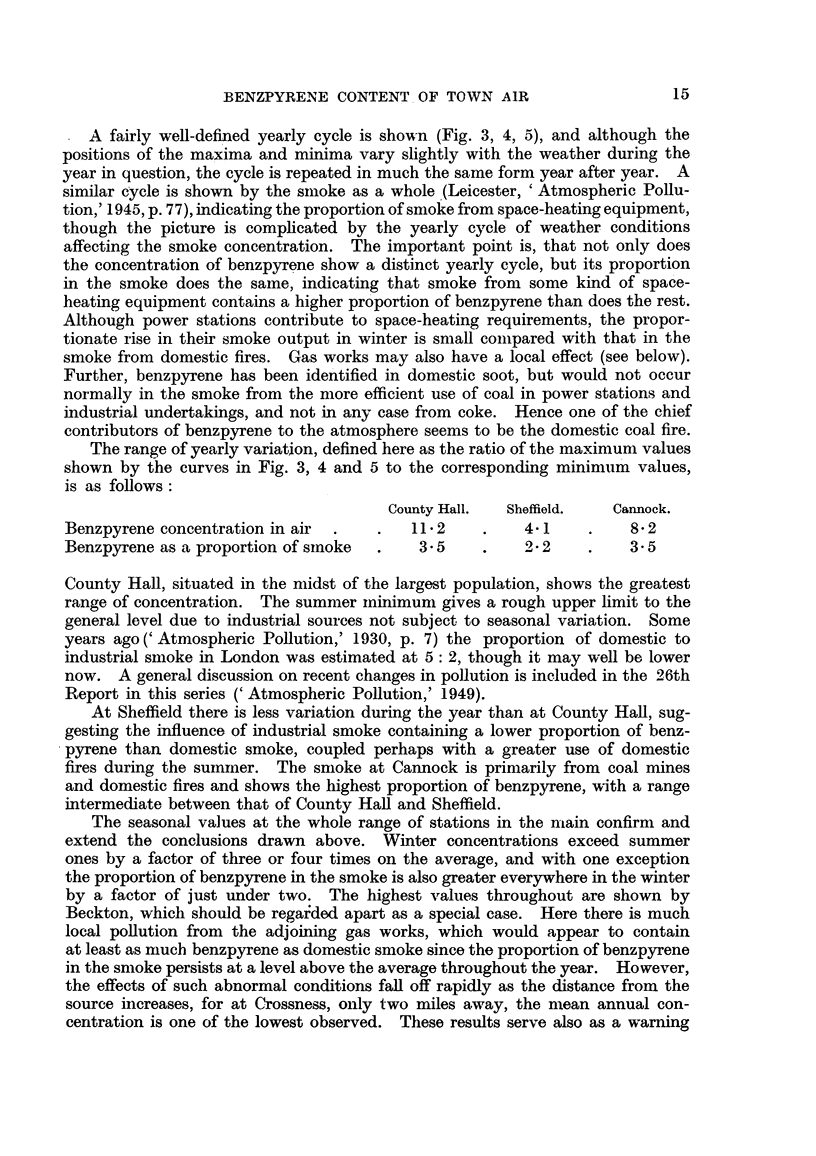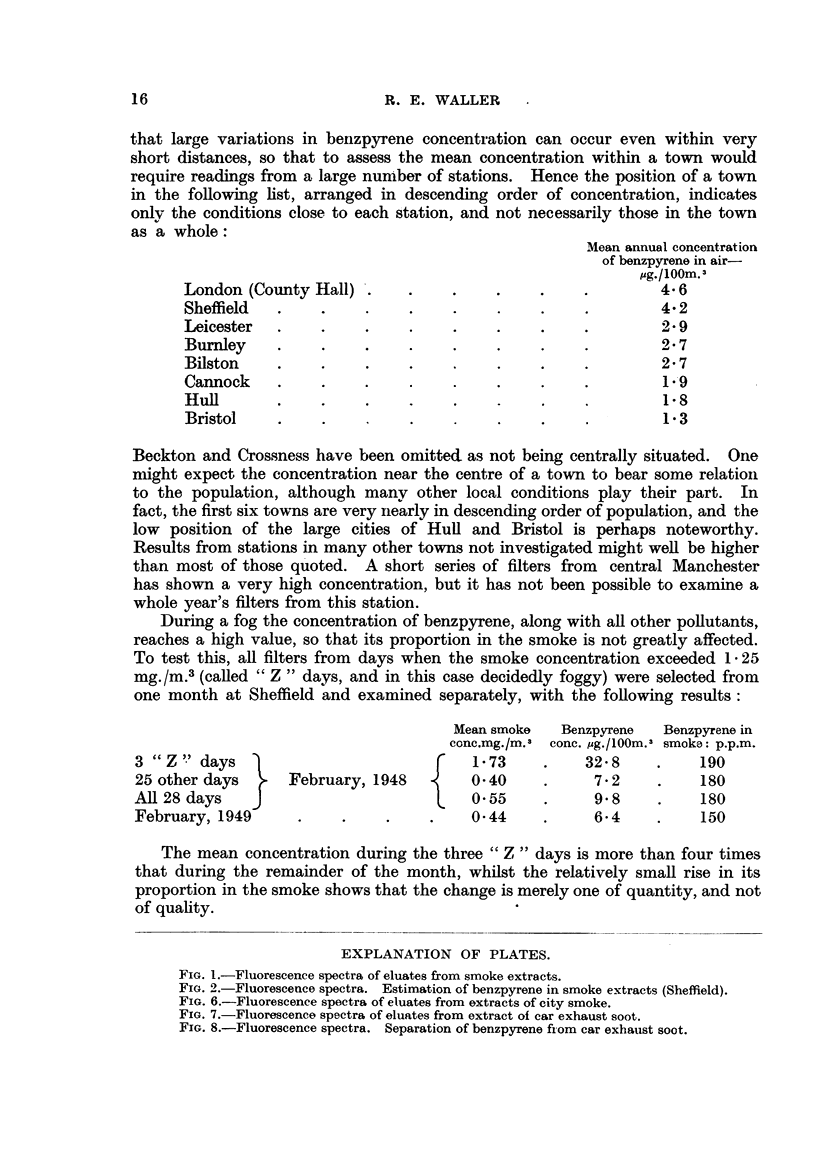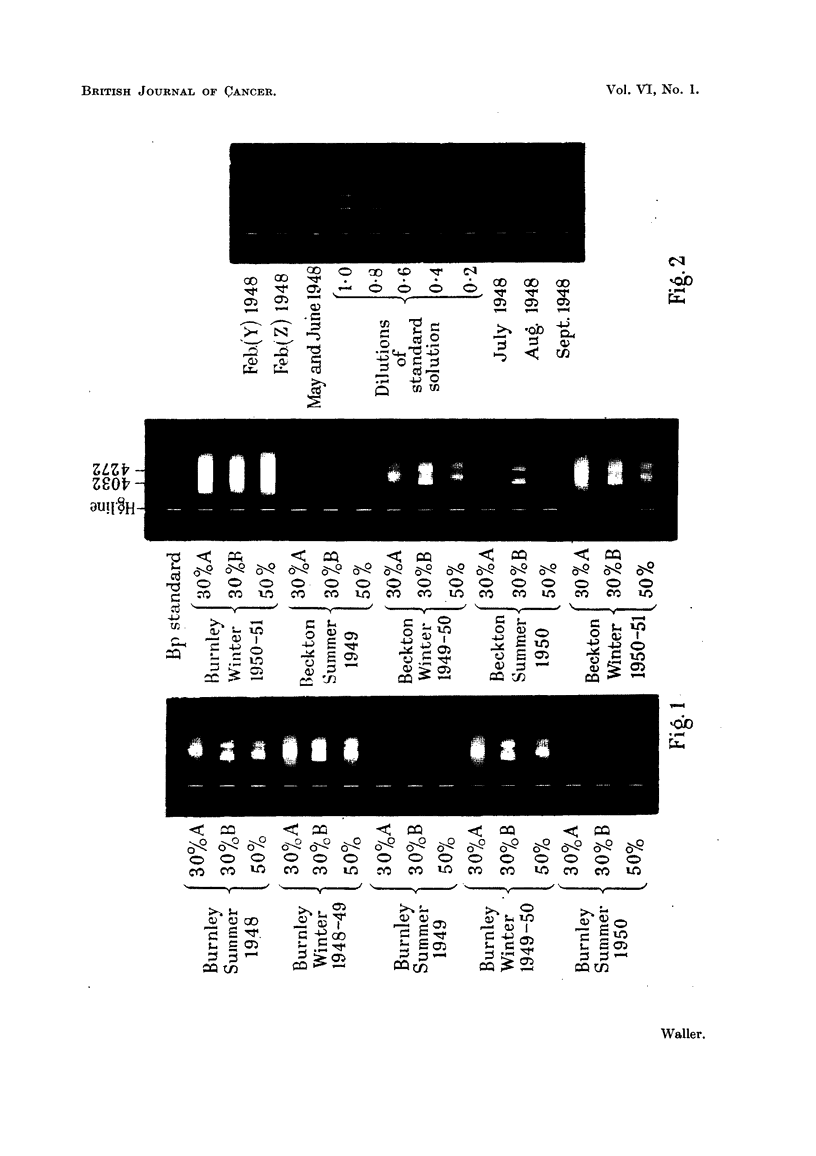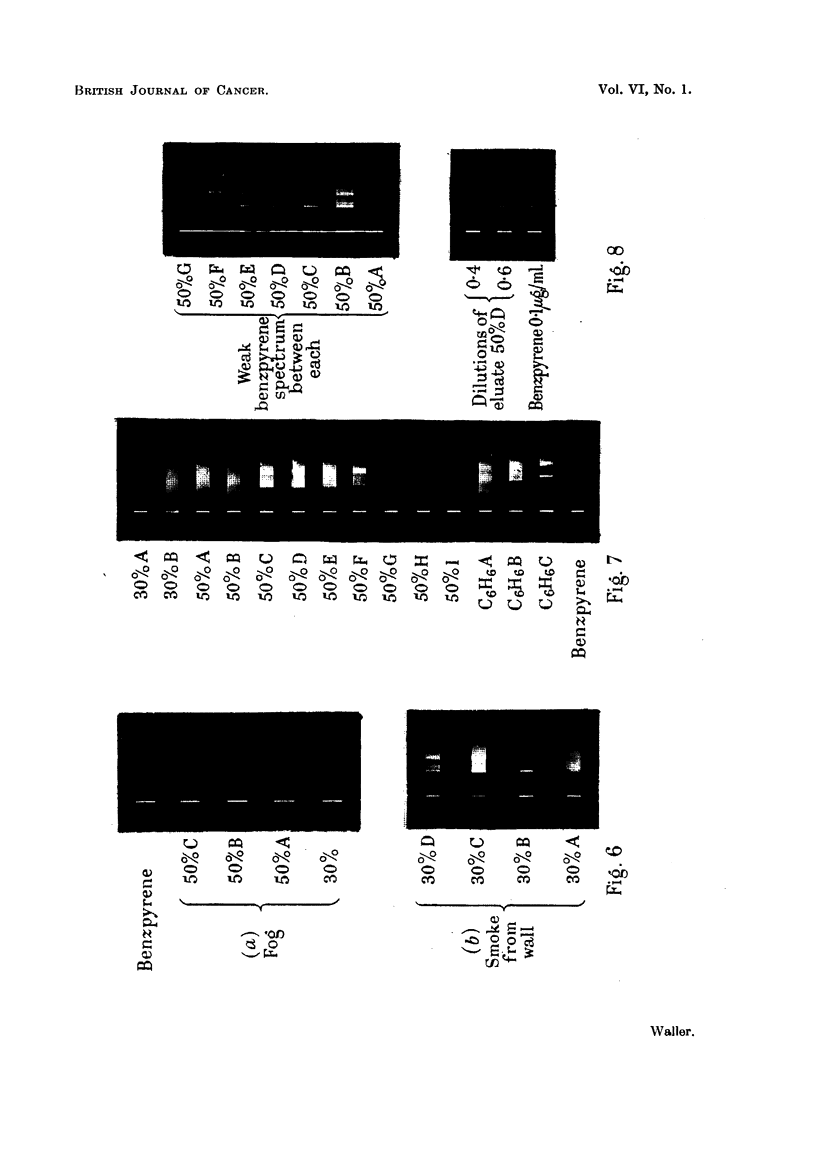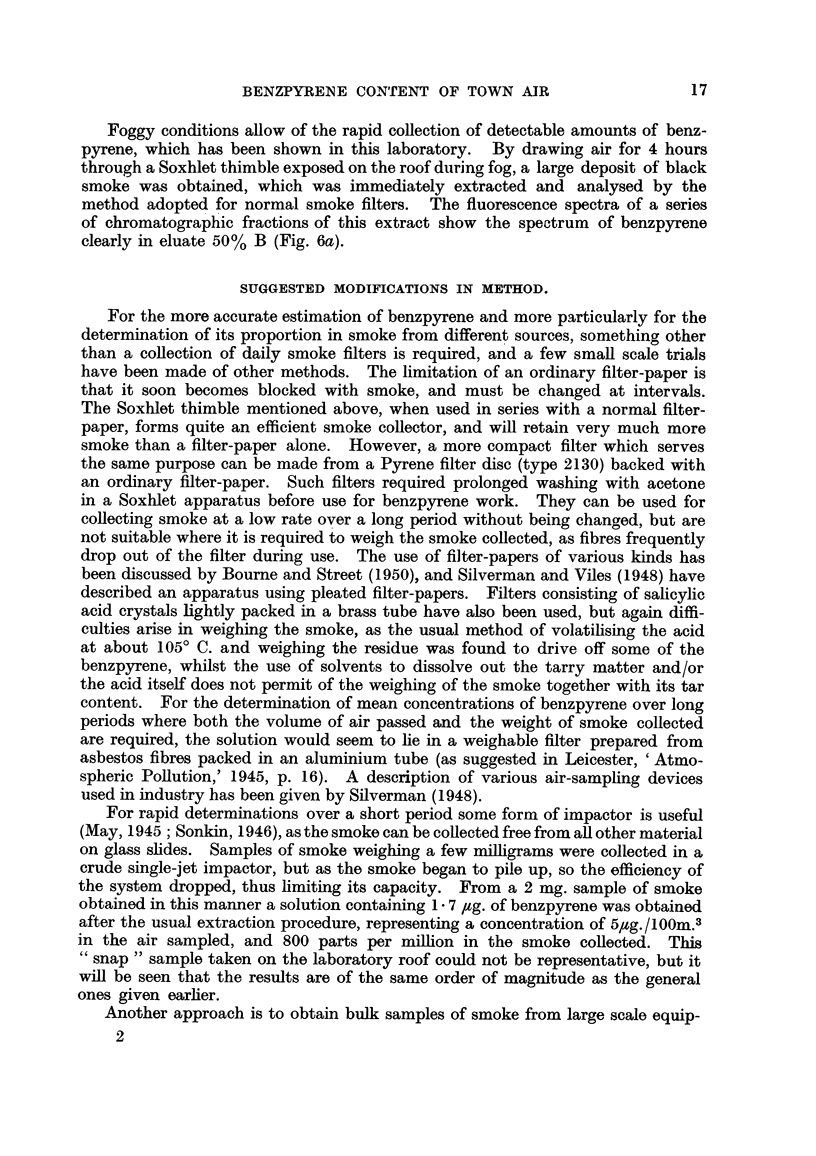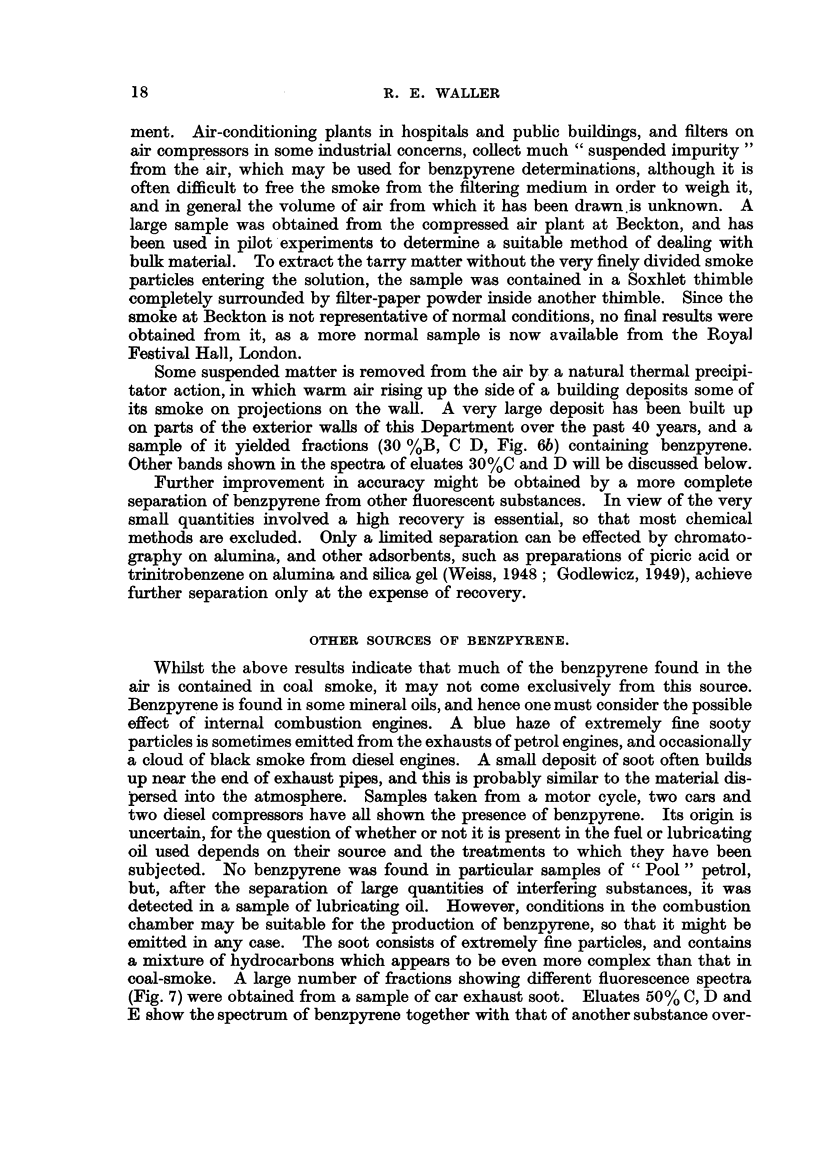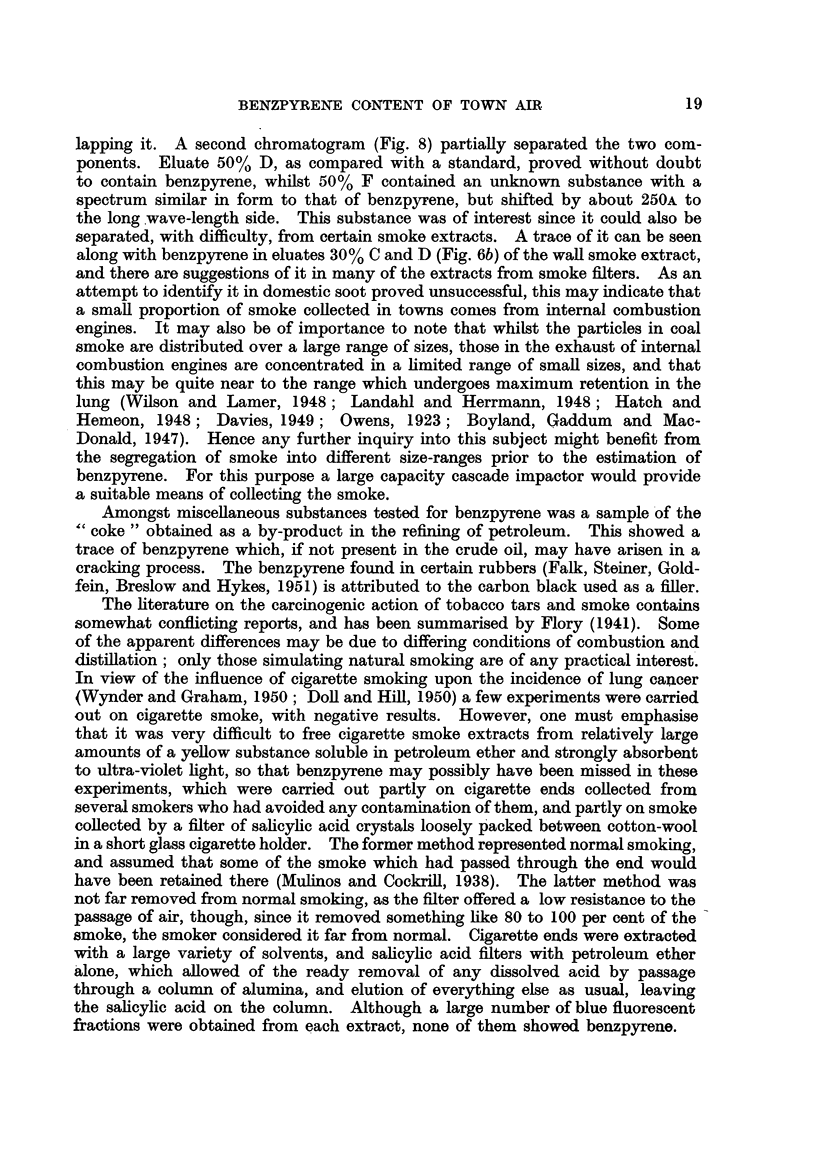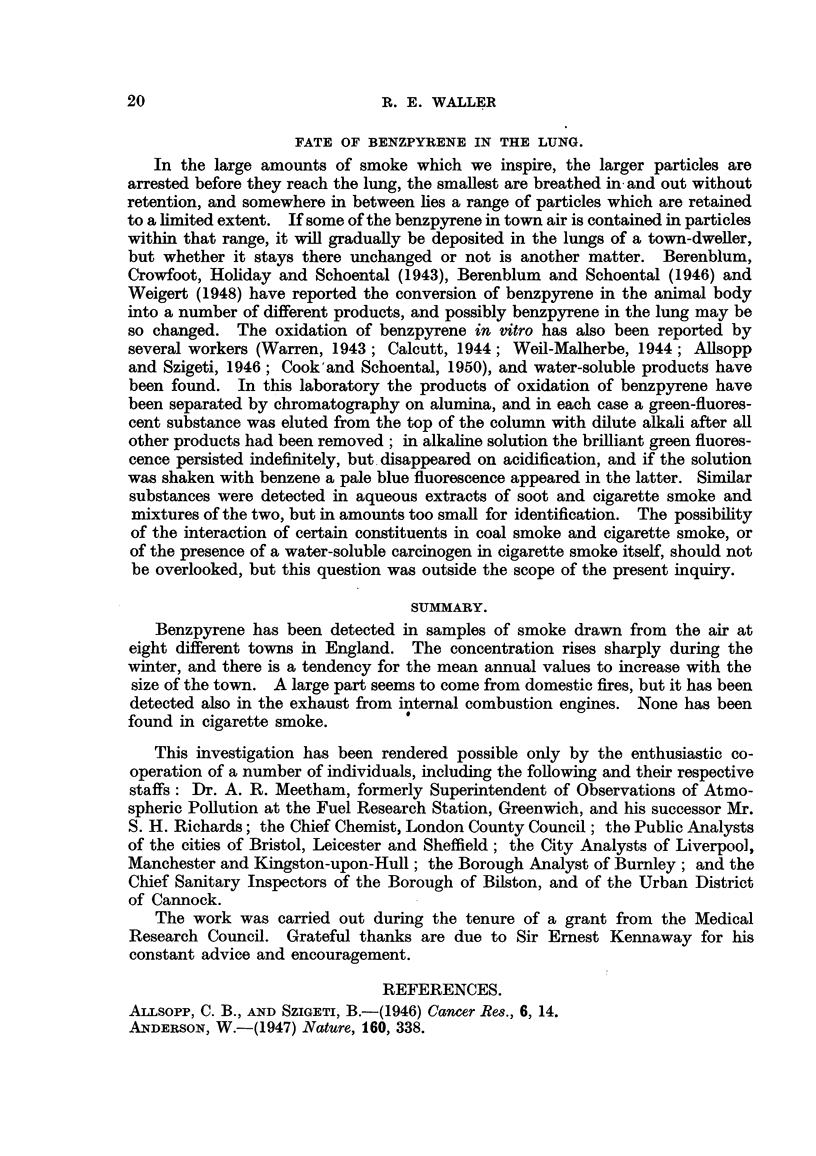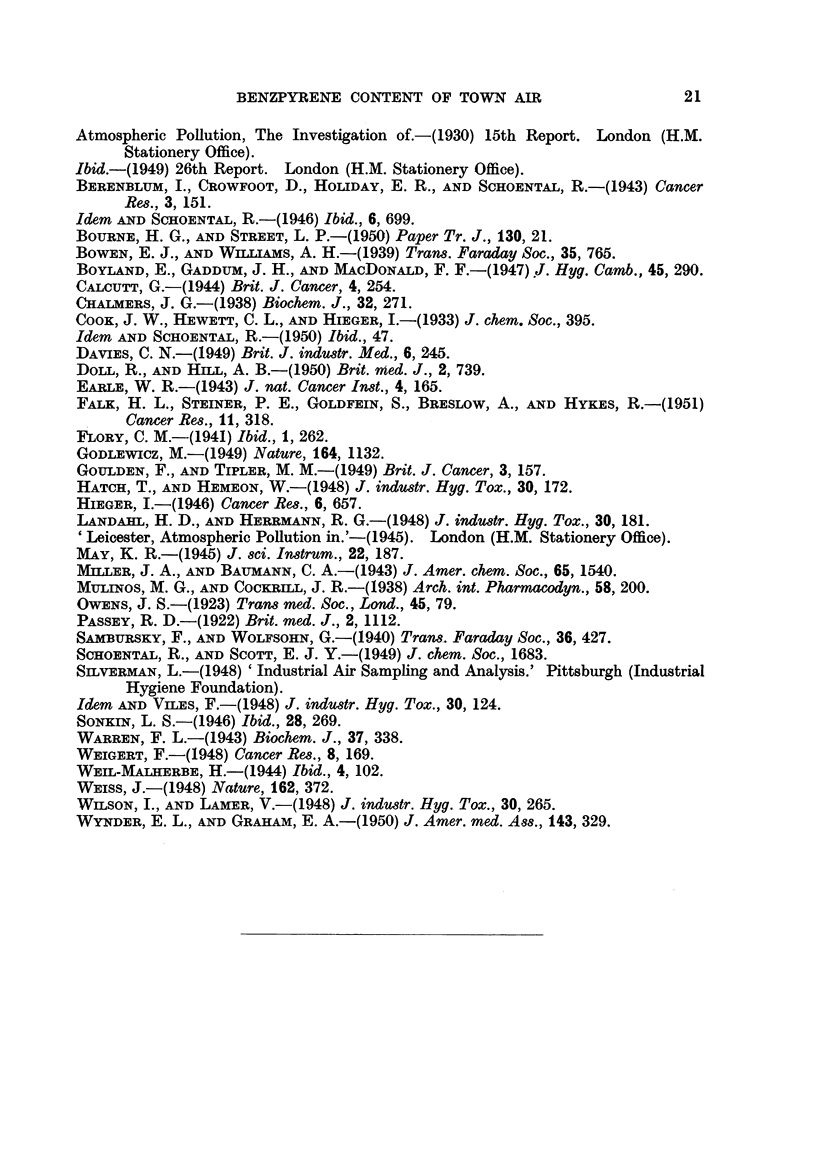# The Benzpyrene Content of Town Air

**DOI:** 10.1038/bjc.1952.2

**Published:** 1952-03

**Authors:** R. E. Waller

## Abstract

**Images:**


### THE BENZPYRENE CONTENT OF TOWN AIR.

R. E. WALLER.

*From the Pathological Department, St. Bartholomew's Hospital, London, E.C.*1.

Received for publication February 11, 1952.

THE use of the fluorescence spectrum by Mayneord and Hieger led to the detection of 3-4-benzpyrene in coal tar and to the isolation of this compound from pitch (Cook, Hewett and Hieger, 1933). Recently Goulden and Tipler (1949) identified it by the same means in domestic soot, which is carcinogenic (Passey, 1922), and their method has been extended to samples of smoke drawn from the atmosphere. By collecting the daily samples taken by local authorities in a number of different areas during several years, it has been possible not only to identify benzpyrene in all samples examined, but to observe its variation with time of year in any one place, to gain an idea of the variation between different places, and to deduce the nature of the principal source of it.

### COLLECTION OF SAMPLES.

Under the guidance of the Department of Scientific and Industrial Research an increasing number of local authorities in large and small towns throughout the country has, during the past few years, collected daily samples of suspended impurity at fairly central points in their respective areas as records of the trends in smoke concentration from month to month and year to year. The apparatus is described fully in " Atmospheric Pollution in Leicester " (Leicester, ' Atmospheric Pollution,' 1945), and generally consists of a small electric motor drawing air from outside a building through a filter-paper, upon which most of the suspended smoke is deposited, followed by a bottle containing $H_2O_2$ for the determination of $SO_2$, and a gas meter to measure the volume of air. Filter-papers are changed daily, and the " shade " of the resulting stain, as compared with a calibrated " scale of shades", is taken as a measure of the smoke collected. These data are collated on monthly sheets showing the daily volume of air sampled, the shade number and smoke concentration deduced therefrom, with monthly summaries. By examination of each monthly set of filters, variations in the quality and quantity of smoke collected at that one station from month to month can be determined. A similar procedure at other stations may enable one, with all due reservations, to make some comparison between them. The smoke consists chiefly of carbon, with some other inorganic matter, and a variable amount of tarry matter, containing a complex mixture of hydrocarbons. In this series of experiments attention was focused on benzpyrene, and no serious attempt was made to identify any other hydrocarbons.

Over 60 stations in Great Britain make observations of atmospheric pollution by this method ; filters from the following ten were used in this investigation.

The figures in brackets after the name of the town give the population in round numbers:

*Bilston* (33,000): on the first floor, in a residential and industrial district. The town lies in a heavily industrialised area with several larger towns nearby.

*Bristol* (442,000): on the second floor, in a busy main thoroughfare, and close to a branch-line railway station and coal yard. Pollution from other towns is unlikely.

*Burnley* (85,000): in the old gas-works yard in the town centre, surrounded by mill chimneys. There are large industrial areas to the south, south-west and east of it.

*Cannock* (41,000): near the centre in a commercial and residential district, with coal mines around the town, which lies just north of a large industrial area. Distance from Bilston 9 miles.

*Hull* (300,000): at about 14 ft. above a main road near the centre of the city, which is well to the east of any other large industrial area.

*Leicester* (285,000): at 40 ft. above a busy road near the centre in a district consisting mainly of offices. The city is fairly well isolated from other industrial areas.

*London* (Beckton): on the first floor at the London County Council Northern Outfall Works just to the south-east of Beckton Gas Works, but fairly isolated from all other buildings, 9 miles east of County Hall.

*London* (County Hall): at 75 ft. above ground level in a commercial district, and roughly at the centre of the Greater London area (population 8,346,000).

*London* (Crossness): near ground level in an open position at the London County Council Southern Outfall Works, with very few adjacent buildings, 11 miles east of County Hall and 2 miles east-south-east of Beckton.

*Sheffield* (513,000): in a manufacturing district in the centre of the city, with factory chimneys nearby.

### PREPARATION OF MATERIALS.

The risk of contamination in the detection of carcinogens has been emphasized by several workers (Earle, 1943; Hieger, 1946; Anderson, 1947). In the present series of estimations of very small quantities of benzpyrene by fluorescence methods the utmost care had to be taken throughout. A high degree of "fluorescence purity" was required of solvents which had to be evaporated down to small volumes. These were redistilled until not only the distillate, but also the residue, showed no fluorescence in ultra-violet light. The fluorescence spectrum of benzpyrene was identified in most first residues from both benzene and petroleum ether. All glass apparatus, together with Soxhlet thimbles and any other equipment, was refluxed for several hours before use with acetone to remove any trace of oil, grease or dust.

### METHOD OF ESTIMATION.

The smoke filters were cut up, packed into thimbles, and extracted in a Soxhlet apparatus with a suitable solvent. In preliminary experiments a mixture of equal parts of benzene and petroleum ether was found to be very satisfactory, as it seemed to extract most of the benzpyrene whilst leaving behind some unwanted substances. However, some of the benzpyrene may be adsorbed upon solid particles in the smoke, and in some cases a second extraction with a more polar solvent extracted up to 50 per cent more benzpyrene. In the main experiment acetone was used, and each extraction was continued until (after about 4 hours) no further fluorescent material could be extracted. The resultant golden-yellow solution was passed through a short column of activated alumina (3 × 1·4 cm., Spence Type H), which removed any finely divided particles, traces of water, and certain of the unwanted hydrocarbons. After running more acetone through the column the resultant solution was evaporated, leaving a yellow oily droplet with a brilliant blue-violet fluorescence in ultra-violet light, which was dissolved as far as possible in several successive lots of boiling petroleum ether (60–80°); the insoluble part was redissolved in acetone and retained separately to be checked for the absence of benzpyrene. The petroleum ether extracts were pooled, evaporated almost to dryness and made up to 20–40 ml. in fresh petroleum ether. This ensured the removal of any trace of acetone left over in the original residue, which was essential for the success of the chromatogram which was to follow. The solution at this stage was pale straw-coloured, with a strong bluish fluorescence in ultra-violet light, and on passing through a column of alumina (8 × 1·4 cm.) previously washed with petroleum ether, most of the fluorescent material was adsorbed as a narrow, blue-fluorescing band near the top of the column.

Development with petroleum ether, followed by a mixture of 30 per cent benzene in petroleum ether, produced a series of rather diffuse bands, distinguishable in ultra-violet light by their slightly differing fluorescence. A pale blue band moved down the column fairly rapidly with the 30 per cent mixture, and was collected as eluate 30% A. This was followed by a blue-violet band collected as 30% B, and a further blue band collected by elution with 50 per cent mixture. After carrying out this procedure on a whole series of smoke filters for various periods and places, the fluorescence spectra of each set of 3 eluates were photographed along with that of a standard solution of pure benzpyrene (Fig. 1).

Solutions were contained in a small glass cell illuminated at 90° by ultra-violet light from a General Electric Co. " Osira " lamp with separate Wood's glass filter, and spectra were recorded on a Hilger E.517 spectrograph, using a 0·1 mm. slit and 2-minute exposures with Ilford " Zenith " plates. All final solutions were prepared in petroleum ether, and no precautions were taken against oxygen quenching (Bowen and Williams, 1939; Miller and Baumann, 1943; Weil-Malherbe, 1944).

Schoental and Scott (1949) give the fluorescence bands of benzpyrene in petroleum ether as a main system at $\lambda$ 4032, 4272 and 4535, with subsidiary systems at $\lambda$ 4082, 4310 and 4149, 4367. With the weak solutions used here, only those at $\lambda$ 4032, 4082, and 4272 appeared in the negatives, and not all of these in reproductions. The group of Hg lines around $\lambda$ 3650 appears in all photographs, and serves as a useful reference line. The position and intensity of the bands varies with the solvent (Chalmers, 1938; Sambursky and Wolfsohn, 1940), but at this stage the difference between petroleum ether and its mixtures with benzene is unimportant. In most cases, the spectrum of benzpyrene was visible in eluate 30% B and not in the others. These former solutions still contained much material other than benzpyrene, some of which fluoresces in the same wave-length region as benzpyrene. Where necessary the solutions were evaporated and rechromatographed, but the benzpyrene was not freed entirely from fluorescent impurities by this means. Finally, each benzpyrene-containing fraction was evaporated and made up in petroleum ether to a known volume. All solutions were examined at a strength such that they fluoresced uniformly across the test cell (i.e., that they were too weak for appreciable re-absorption to occur). To estimate the amount of benzpyrene in each solution, a control experiment was made in which a known amount of benzpyrene was mixed with non-benzpyrene-containing impurities recovered from the main experiment and the whole procedure repeated. By preparing a series of successive dilutions from the final solution of this experiment, a set of standards was obtained with which the unknowns could be compared. The spectra of all solutions were then photographed together on one plate (Fig. 2) and the intensity of each unknown matched against one of the standards by visual comparison. The total amount of benzpyrene extracted from each set of smoke filters could thus be calculated, and hence its concentration in the air sampled, and proportion in the smoke collected, deduced. This method compensates for the slight loss of benzpyrene during the experiment, for a control on pure benzpyrene alone showed that the recovery was of the order of 80 per cent. A blank experiment, substituting the clean surrounds of the filter-papers for their black centres showed a trace of benzpyrene, but too little to estimate. Apart from extreme sensitivity to contaminants in working materials, the method proved consistent in a large number of estimations and controls carried out at different times, so that although the absolute values of the results cannot be guaranteed, comparisons between them should be valid.

### RESULTS.

After trials to determine the most suitable methods and periods, the remaining smoke filters were dealt with in three groups, as follows :

(i) A monthly survey for 12 consecutive months at London (County Hall), Sheffield and Cannock, chosen to represent towns of greatly differing size (Table I).

TABLE I.—*Monthly Concentrations of Benzpyrene.*

| | Concentration in air, µg./100m.³ | | | Proportion in smoke, parts per million. | | |
|---|---|---|---|---|---|---|
| | London (County Hall). | Sheffield. | Cannock. | London (County Hall). | Sheffield. | Cannock. |
| May, 1949 | — | 2·0 (May–June) | — | — | 70 (May–June) | — |
| June, ,, | 2·6 | | — | 140 | | — |
| July, ,, | 1·2 | 2·4 | 0·6 (July–Aug.) | 75 | 80 | 120 (July–Aug.) |
| August, ,, | 2·1 | 2·1 | | 150 | 80 | |
| September, ,, | 1·4 | 3·3 | 1·1 | 80 | 100 | 150 |
| October, ,, | 4·4 | 5·8 | 2·7 | 150 | 130 | 290 |
| November, ,, | 8·0 | 5·6 | 3·1 | 160 | 130 | 290 |
| December ,, | 12·2 | 6·3 | 2·7 | 280 | 150 | 360 |
| January, 1950 | 14·7 | 7·8 | 3·2 | 330 | 150 | 350 |
| February, ,, | 9·5 | 6·4 | 2·7 | 280 | 150 | 340 |
| March, ,, | 10·1 | 6·5 | 2·7 | 290 | 140 | 440 |
| April, ,, | 4·8 | 4·4 | 1·1 | 240 | 130 | 370 |
| May, ,, | 2·5 | — | 1·6 | 160 | — | 260 |
| June, ,, | — | — | 0·4 | — | — | 125 |

(ii) A survey over 3 consecutive years at London (Crossness), as a check on year-to-year variations (Table II).

TABLE II.—*Seasonal and Mean Annual Concentrations of Benzpyrene.*

*Concentration in Air: µg./100m.³*

| | | Bilston. | Bristol. | Burnley. | Cannock. | Hull. | Leicester. | London. Beckton. | London. County Hall. | London. Crossness. | Sheffield. |
|---|---|---|---|---|---|---|---|---|---|---|---|
| Seasonal values | Summer, 1948 | — | — | 1·3 | — | — | — | — | — | — | — |
| | Winter, 1948–9 | — | — | 4·8 | — | — | — | — | — | — | — |
| | Summer, 1949 | — | — | 1·4 | 0·9† | — | — | 2·5 | 2·0 | — | 2·3 |
| | Winter, 1949–50 | — | — | 3·5 | 2·9 | 2·5 | — | 12·2 | 10·4 | — | 6·5 |
| | Summer, 1950 | 0·8 | 0·5 | 1·3 | — | 0·9 | — | 4·8 | 1·3 | — | 1·8 |
| | Winter, 1950–51 | 3·5 | 2·1* | 4·0 | 2·1 | 2·7 | 3·5 | 9·1 | 4·5 | — | 6·1 |
| Ratio of winter to summer values | 1948–49 | — | — | 3·7 | — | — | — | — | — | — | — |
| | 1949–50 | — | — | 2·5 | 3·2 | — | — | 4·9 | 5·2 | — | 2·8 |
| | 1950–51 | 4·4 | 4·2 | 3·1 | — | 3·0 | — | 1·9 | 3·5 | — | 3·4 |
| | Mean | 4·4 | 4·2 | 3·1 | 3·2 | 3·0 | — | 3·4 | 4·3 | — | 3·1 |
| Mean annual values | May, 1947–Apr., 1948 | — | — | — | — | — | — | — | — | 1·7 | — |
| | May, 1948–Apr., 1949 | 3·2‡ | — | 3·0 | — | — | 2·9§ | — | — | 1·6 | — |
| | May, 1949–Apr., 1950 | — | — | 2·5 | 1·9 | — | — | 7·3 | 6·2 | 1·5 | 4·4 |
| | May, 1950–Apr., 1951 | 2·2 | 1·3 | 2·7 | — | 1·8 | — | 7·0 | 2·9 | — | 4·0 |
| | Mean | 2·7 | 1·3 | 2·7 | 1·9 | 1·8 | 2·9 | 7·1 | 4·6 | 1·6 | 4·2 |

*Proportion in Smoke: parts per million*

| | | Bilston. | Bristol. | Burnley. | Cannock. | Hull. | Leicester. | London. Beckton. | London. County Hall. | London. Crossness. | Sheffield. |
|---|---|---|---|---|---|---|---|---|---|---|---|
| Seasonal values | Summer, 1948 | — | — | 56 | — | — | — | — | — | — | — |
| | Winter, 1948–9 | — | — | 116 | — | — | — | — | — | — | — |
| | Summer, 1949 | — | — | 59 | 167† | — | — | 185 | 123 | — | 81 |
| | Winter, 1949–50 | — | — | 92 | 352 | 82 | — | 284 | 270 | — | 143 |
| | Summer, 1950 | 54 | 146 | 71 | — | 50 | — | 268 | 108 | — | 83 |
| | Winter, 1950–51 | 94 | 242* | 141 | 140 | 69 | 139 | 234 | 123 | — | 120 |
| Ratio of winter to summer values | 1948–49 | — | — | 2·1 | — | — | — | — | — | — | — |
| | 1949–50 | — | — | 1·6 | 2·1 | — | — | 1·5 | 2·2 | — | 1·8 |
| | 1950–51 | 1·7 | 1·7 | 2·0 | — | 1·4 | — | 0·9 | 1·1 | — | 1·4 |
| | Mean | 1·7 | 1·7 | 1·9 | 2·1 | 1·4 | — | 1·2 | 1·7 | — | 1·6 |
| Mean annual values | May, 1947–Apr., 1948 | — | — | — | — | — | — | — | — | — | — |
| | May, 1948–Apr., 1949 | 105‡ | — | 86 | — | — | 205§ | — | — | — | — |
| | May, 1949–Apr., 1950 | — | — | 76 | 260 | — | — | 235 | 196 | — | 112 |
| | May, 1950–Apr., 1951 | 74 | 194 | 106 | — | 60 | — | 251 | 116 | — | 102 |
| | Mean | 90 | 194 | 89 | 260 | 60 | 205 | 243 | 156 | — | 107 |

* March, 1950, substituted for March, 1951.
† May and June, 1950, substituted for May and June, 1949.
‡ Twelve month period, April, 1948, to March, 1949.
§ Twelve month period, October, 1948, to September, 1949.

(iii) A seasonal survey over as many summers and winters as possible at all remaining stations ; summer is defined as the five months May to September, and winter as November to March ; the mean of the two periods is taken as approximately the mean annual value (Table II).

FIG. 3.—Yearly cycle of benzpyrene at London (County Hall).

The variations from month to month at any one station are greater than the difference between stations, so that a mean value over at least one year is required for any comparisons. Table II shows appreciable variations from one year to the next ; an average over something like 5 years would be required to give reliable figures. As this is impracticable at present, mean annual values (Table II) representing averages over 1 to 3 years, must be taken as subject to a large margin of error.

FIG. 4.—Yearly cycle of benzpyrene at Sheffield.

FIG. 5.—Yearly cycle of benzpyrene at Cannock.

A fairly well-defined yearly cycle is shown (Fig. 3, 4, 5), and although the positions of the maxima and minima vary slightly with the weather during the year in question, the cycle is repeated in much the same form year after year. A similar cycle is shown by the smoke as a whole (Leicester, 'Atmospheric Pollution,' 1945, p. 77), indicating the proportion of smoke from space-heating equipment, though the picture is complicated by the yearly cycle of weather conditions affecting the smoke concentration. The important point is, that not only does the concentration of benzpyrene show a distinct yearly cycle, but its proportion in the smoke does the same, indicating that smoke from some kind of space-heating equipment contains a higher proportion of benzpyrene than does the rest. Although power stations contribute to space-heating requirements, the proportionate rise in their smoke output in winter is small compared with that in the smoke from domestic fires. Gas works may also have a local effect (see below). Further, benzpyrene has been identified in domestic soot, but would not occur normally in the smoke from the more efficient use of coal in power stations and industrial undertakings, and not in any case from coke. Hence one of the chief contributors of benzpyrene to the atmosphere seems to be the domestic coal fire.

The range of yearly variation, defined here as the ratio of the maximum values shown by the curves in Fig. 3, 4 and 5 to the corresponding minimum values, is as follows :

| | County Hall. | Sheffield. | Cannock. |
|---|---|---|---|
| Benzpyrene concentration in air | 11·2 | 4·1 | 8·2 |
| Benzpyrene as a proportion of smoke | 3·5 | 2·2 | 3·5 |

County Hall, situated in the midst of the largest population, shows the greatest range of concentration. The summer minimum gives a rough upper limit to the general level due to industrial sources not subject to seasonal variation. Some years ago ('Atmospheric Pollution,' 1930, p. 7) the proportion of domestic to industrial smoke in London was estimated at 5 : 2, though it may well be lower now. A general discussion on recent changes in pollution is included in the 26th Report in this series ('Atmospheric Pollution,' 1949).

At Sheffield there is less variation during the year than at County Hall, suggesting the influence of industrial smoke containing a lower proportion of benzpyrene than domestic smoke, coupled perhaps with a greater use of domestic fires during the summer. The smoke at Cannock is primarily from coal mines and domestic fires and shows the highest proportion of benzpyrene, with a range intermediate between that of County Hall and Sheffield.

The seasonal values at the whole range of stations in the main confirm and extend the conclusions drawn above. Winter concentrations exceed summer ones by a factor of three or four times on the average, and with one exception the proportion of benzpyrene in the smoke is also greater everywhere in the winter by a factor of just under two. The highest values throughout are shown by Beckton, which should be regarded apart as a special case. Here there is much local pollution from the adjoining gas works, which would appear to contain at least as much benzpyrene as domestic smoke since the proportion of benzpyrene in the smoke persists at a level above the average throughout the year. However, the effects of such abnormal conditions fall off rapidly as the distance from the source increases, for at Crossness, only two miles away, the mean annual concentration is one of the lowest observed. These results serve also as a warning that large variations in benzpyrene concentration can occur even within very short distances, so that to assess the mean concentration within a town would require readings from a large number of stations. Hence the position of a town in the following list, arranged in descending order of concentration, indicates only the conditions close to each station, and not necessarily those in the town as a whole:

| | Mean annual concentration of benzpyrene in air—$\mu$g./100m.$^3$ |
|---|---|
| London (County Hall) | 4·6 |
| Sheffield | 4·2 |
| Leicester | 2·9 |
| Burnley | 2·7 |
| Bilston | 2·7 |
| Cannock | 1·9 |
| Hull | 1·8 |
| Bristol | 1·3 |

Beckton and Crossness have been omitted as not being centrally situated. One might expect the concentration near the centre of a town to bear some relation to the population, although many other local conditions play their part. In fact, the first six towns are very nearly in descending order of population, and the low position of the large cities of Hull and Bristol is perhaps noteworthy. Results from stations in many other towns not investigated might well be higher than most of those quoted. A short series of filters from central Manchester has shown a very high concentration, but it has not been possible to examine a whole year's filters from this station.

During a fog the concentration of benzpyrene, along with all other pollutants, reaches a high value, so that its proportion in the smoke is not greatly affected. To test this, all filters from days when the smoke concentration exceeded 1·25 mg./m.$^3$ (called " Z " days, and in this case decidedly foggy) were selected from one month at Sheffield and examined separately, with the following results:

| | | Mean smoke conc.mg./m.$^3$ | Benzpyrene conc. $\mu$g./100m.$^3$ | Benzpyrene in smoke: p.p.m. |
|---|---|---|---|---|
| 3 " Z " days | February, 1948 | 1·73 | 32·8 | 190 |
| 25 other days | February, 1948 | 0·40 | 7·2 | 180 |
| All 28 days | February, 1948 | 0·55 | 9·8 | 180 |
| February, 1949 | | 0·44 | 6·4 | 150 |

The mean concentration during the three " Z " days is more than four times that during the remainder of the month, whilst the relatively small rise in its proportion in the smoke shows that the change is merely one of quantity, and not of quality.

### EXPLANATION OF PLATES.

FIG. 1.—Fluorescence spectra of eluates from smoke extracts.
FIG. 2.—Fluorescence spectra. Estimation of benzpyrene in smoke extracts (Sheffield).
FIG. 6.—Fluorescence spectra of eluates from extracts of city smoke.
FIG. 7.—Fluorescence spectra of eluates from extract of car exhaust soot.
FIG. 8.—Fluorescence spectra. Separation of benzpyrene from car exhaust soot.

Fig. 1

Fig. 2

Waller.

Fig. 6

Fig. 7

Fig. 8

Foggy conditions allow of the rapid collection of detectable amounts of benzpyrene, which has been shown in this laboratory. By drawing air for 4 hours through a Soxhlet thimble exposed on the roof during fog, a large deposit of black smoke was obtained, which was immediately extracted and analysed by the method adopted for normal smoke filters. The fluorescence spectra of a series of chromatographic fractions of this extract show the spectrum of benzpyrene clearly in eluate 50% B (Fig. 6*a*).

### SUGGESTED MODIFICATIONS IN METHOD.

For the more accurate estimation of benzpyrene and more particularly for the determination of its proportion in smoke from different sources, something other than a collection of daily smoke filters is required, and a few small scale trials have been made of other methods. The limitation of an ordinary filter-paper is that it soon becomes blocked with smoke, and must be changed at intervals. The Soxhlet thimble mentioned above, when used in series with a normal filter-paper, forms quite an efficient smoke collector, and will retain very much more smoke than a filter-paper alone. However, a more compact filter which serves the same purpose can be made from a Pyrene filter disc (type 2130) backed with an ordinary filter-paper. Such filters required prolonged washing with acetone in a Soxhlet apparatus before use for benzpyrene work. They can be used for collecting smoke at a low rate over a long period without being changed, but are not suitable where it is required to weigh the smoke collected, as fibres frequently drop out of the filter during use. The use of filter-papers of various kinds has been discussed by Bourne and Street (1950), and Silverman and Viles (1948) have described an apparatus using pleated filter-papers. Filters consisting of salicylic acid crystals lightly packed in a brass tube have also been used, but again difficulties arise in weighing the smoke, as the usual method of volatilising the acid at about 105° C. and weighing the residue was found to drive off some of the benzpyrene, whilst the use of solvents to dissolve out the tarry matter and/or the acid itself does not permit of the weighing of the smoke together with its tar content. For the determination of mean concentrations of benzpyrene over long periods where both the volume of air passed and the weight of smoke collected are required, the solution would seem to lie in a weighable filter prepared from asbestos fibres packed in an aluminium tube (as suggested in Leicester, 'Atmospheric Pollution,' 1945, p. 16). A description of various air-sampling devices used in industry has been given by Silverman (1948).

For rapid determinations over a short period some form of impactor is useful (May, 1945 ; Sonkin, 1946), as the smoke can be collected free from all other material on glass slides. Samples of smoke weighing a few milligrams were collected in a crude single-jet impactor, but as the smoke began to pile up, so the efficiency of the system dropped, thus limiting its capacity. From a 2 mg. sample of smoke obtained in this manner a solution containing 1·7 $\mu$g. of benzpyrene was obtained after the usual extraction procedure, representing a concentration of 5$\mu$g./100m.$^3$ in the air sampled, and 800 parts per million in the smoke collected. This "snap" sample taken on the laboratory roof could not be representative, but it will be seen that the results are of the same order of magnitude as the general ones given earlier.

Another approach is to obtain bulk samples of smoke from large scale equipment. Air-conditioning plants in hospitals and public buildings, and filters on air compressors in some industrial concerns, collect much " suspended impurity " from the air, which may be used for benzpyrene determinations, although it is often difficult to free the smoke from the filtering medium in order to weigh it, and in general the volume of air from which it has been drawn is unknown. A large sample was obtained from the compressed air plant at Beckton, and has been used in pilot experiments to determine a suitable method of dealing with bulk material. To extract the tarry matter without the very finely divided smoke particles entering the solution, the sample was contained in a Soxhlet thimble completely surrounded by filter-paper powder inside another thimble. Since the smoke at Beckton is not representative of normal conditions, no final results were obtained from it, as a more normal sample is now available from the Royal Festival Hall, London.

Some suspended matter is removed from the air by a natural thermal precipitator action, in which warm air rising up the side of a building deposits some of its smoke on projections on the wall. A very large deposit has been built up on parts of the exterior walls of this Department over the past 40 years, and a sample of it yielded fractions (30 %B, C D, Fig. 6*b*) containing benzpyrene. Other bands shown in the spectra of eluates 30%C and D will be discussed below.

Further improvement in accuracy might be obtained by a more complete separation of benzpyrene from other fluorescent substances. In view of the very small quantities involved a high recovery is essential, so that most chemical methods are excluded. Only a limited separation can be effected by chromatography on alumina, and other adsorbents, such as preparations of picric acid or trinitrobenzene on alumina and silica gel (Weiss, 1948 ; Godlewicz, 1949), achieve further separation only at the expense of recovery.

### OTHER SOURCES OF BENZPYRENE.

Whilst the above results indicate that much of the benzpyrene found in the air is contained in coal smoke, it may not come exclusively from this source. Benzpyrene is found in some mineral oils, and hence one must consider the possible effect of internal combustion engines. A blue haze of extremely fine sooty particles is sometimes emitted from the exhausts of petrol engines, and occasionally a cloud of black smoke from diesel engines. A small deposit of soot often builds up near the end of exhaust pipes, and this is probably similar to the material dispersed into the atmosphere. Samples taken from a motor cycle, two cars and two diesel compressors have all shown the presence of benzpyrene. Its origin is uncertain, for the question of whether or not it is present in the fuel or lubricating oil used depends on their source and the treatments to which they have been subjected. No benzpyrene was found in particular samples of " Pool " petrol, but, after the separation of large quantities of interfering substances, it was detected in a sample of lubricating oil. However, conditions in the combustion chamber may be suitable for the production of benzpyrene, so that it might be emitted in any case. The soot consists of extremely fine particles, and contains a mixture of hydrocarbons which appears to be even more complex than that in coal-smoke. A large number of fractions showing different fluorescence spectra (Fig. 7) were obtained from a sample of car exhaust soot. Eluates 50% C, D and E show the spectrum of benzpyrene together with that of another substance overlapping it. A second chromatogram (Fig. 8) partially separated the two components. Eluate 50% D, as compared with a standard, proved without doubt to contain benzpyrene, whilst 50% F contained an unknown substance with a spectrum similar in form to that of benzpyrene, but shifted by about 250A to the long wave-length side. This substance was of interest since it could also be separated, with difficulty, from certain smoke extracts. A trace of it can be seen along with benzpyrene in eluates 30% C and D (Fig. 6*b*) of the wall smoke extract, and there are suggestions of it in many of the extracts from smoke filters. As an attempt to identify it in domestic soot proved unsuccessful, this may indicate that a small proportion of smoke collected in towns comes from internal combustion engines. It may also be of importance to note that whilst the particles in coal smoke are distributed over a large range of sizes, those in the exhaust of internal combustion engines are concentrated in a limited range of small sizes, and that this may be quite near to the range which undergoes maximum retention in the lung (Wilson and Lamer, 1948 ; Landahl and Herrmann, 1948 ; Hatch and Hemeon, 1948 ; Davies, 1949 ; Owens, 1923 ; Boyland, Gaddum and MacDonald, 1947). Hence any further inquiry into this subject might benefit from the segregation of smoke into different size-ranges prior to the estimation of benzpyrene. For this purpose a large capacity cascade impactor would provide a suitable means of collecting the smoke.

Amongst miscellaneous substances tested for benzpyrene was a sample of the " coke " obtained as a by-product in the refining of petroleum. This showed a trace of benzpyrene which, if not present in the crude oil, may have arisen in a cracking process. The benzpyrene found in certain rubbers (Falk, Steiner, Goldfein, Breslow and Hykes, 1951) is attributed to the carbon black used as a filler.

The literature on the carcinogenic action of tobacco tars and smoke contains somewhat conflicting reports, and has been summarised by Flory (1941). Some of the apparent differences may be due to differing conditions of combustion and distillation ; only those simulating natural smoking are of any practical interest. In view of the influence of cigarette smoking upon the incidence of lung cancer (Wynder and Graham, 1950 ; Doll and Hill, 1950) a few experiments were carried out on cigarette smoke, with negative results. However, one must emphasise that it was very difficult to free cigarette smoke extracts from relatively large amounts of a yellow substance soluble in petroleum ether and strongly absorbent to ultra-violet light, so that benzpyrene may possibly have been missed in these experiments, which were carried out partly on cigarette ends collected from several smokers who had avoided any contamination of them, and partly on smoke collected by a filter of salicylic acid crystals loosely packed between cotton-wool in a short glass cigarette holder. The former method represented normal smoking, and assumed that some of the smoke which had passed through the end would have been retained there (Mulinos and Cockrill, 1938). The latter method was not far removed from normal smoking, as the filter offered a low resistance to the passage of air, though, since it removed something like 80 to 100 per cent of the smoke, the smoker considered it far from normal. Cigarette ends were extracted with a large variety of solvents, and salicylic acid filters with petroleum ether alone, which allowed of the ready removal of any dissolved acid by passage through a column of alumina, and elution of everything else as usual, leaving the salicylic acid on the column. Although a large number of blue fluorescent fractions were obtained from each extract, none of them showed benzpyrene.

FATE OF BENZPYRENE IN THE LUNG.

In the large amounts of smoke which we inspire, the larger particles are arrested before they reach the lung, the smallest are breathed in and out without retention, and somewhere in between lies a range of particles which are retained to a limited extent. If some of the benzpyrene in town air is contained in particles within that range, it will gradually be deposited in the lungs of a town-dweller, but whether it stays there unchanged or not is another matter. Berenblum, Crowfoot, Holiday and Schoental (1943), Berenblum and Schoental (1946) and Weigert (1948) have reported the conversion of benzpyrene in the animal body into a number of different products, and possibly benzpyrene in the lung may be so changed. The oxidation of benzpyrene *in vitro* has also been reported by several workers (Warren, 1943; Calcutt, 1944; Weil-Malherbe, 1944; Allsopp and Szigeti, 1946; Cook and Schoental, 1950), and water-soluble products have been found. In this laboratory the products of oxidation of benzpyrene have been separated by chromatography on alumina, and in each case a green-fluorescent substance was eluted from the top of the column with dilute alkali after all other products had been removed; in alkaline solution the brilliant green fluorescence persisted indefinitely, but disappeared on acidification, and if the solution was shaken with benzene a pale blue fluorescence appeared in the latter. Similar substances were detected in aqueous extracts of soot and cigarette smoke and mixtures of the two, but in amounts too small for identification. The possibility of the interaction of certain constituents in coal smoke and cigarette smoke, or of the presence of a water-soluble carcinogen in cigarette smoke itself, should not be overlooked, but this question was outside the scope of the present inquiry.

SUMMARY.

Benzpyrene has been detected in samples of smoke drawn from the air at eight different towns in England. The concentration rises sharply during the winter, and there is a tendency for the mean annual values to increase with the size of the town. A large part seems to come from domestic fires, but it has been detected also in the exhaust from internal combustion engines. None has been found in cigarette smoke.

This investigation has been rendered possible only by the enthusiastic co-operation of a number of individuals, including the following and their respective staffs: Dr. A. R. Meetham, formerly Superintendent of Observations of Atmospheric Pollution at the Fuel Research Station, Greenwich, and his successor Mr. S. H. Richards; the Chief Chemist, London County Council; the Public Analysts of the cities of Bristol, Leicester and Sheffield; the City Analysts of Liverpool, Manchester and Kingston-upon-Hull; the Borough Analyst of Burnley; and the Chief Sanitary Inspectors of the Borough of Bilston, and of the Urban District of Cannock.

The work was carried out during the tenure of a grant from the Medical Research Council. Grateful thanks are due to Sir Ernest Kennaway for his constant advice and encouragement.

REFERENCES.

Allsopp, C. B., and Szigeti, B.—(1946) *Cancer Res.*, **6**, 14.
Anderson, W.—(1947) *Nature*, **160**, 338.

Atmospheric Pollution, The Investigation of.—(1930) 15th Report. London (H.M. Stationery Office).
*Ibid.*—(1949) 26th Report. London (H.M. Stationery Office).
BERENBLUM, I., CROWFOOT, D., HOLIDAY, E. R., AND SCHOENTAL, R.—(1943) *Cancer Res.*, **3**, 151.
*Idem* AND SCHOENTAL, R.—(1946) *Ibid.*, **6**, 699.
BOURNE, H. G., AND STREET, L. P.—(1950) *Paper Tr. J.*, **130**, 21.
BOWEN, E. J., AND WILLIAMS, A. H.—(1939) *Trans. Faraday Soc.*, **35**, 765.
BOYLAND, E., GADDUM, J. H., AND MACDONALD, F. F.—(1947) *J. Hyg. Camb.*, **45**, 290.
CALCUTT, G.—(1944) *Brit. J. Cancer*, **4**, 254.
CHALMERS, J. G.—(1938) *Biochem. J.*, **32**, 271.
COOK, J. W., HEWETT, C. L., AND HIEGER, I.—(1933) *J. chem. Soc.*, 395.
*Idem* AND SCHOENTAL, R.—(1950) *Ibid.*, 47.
DAVIES, C. N.—(1949) *Brit. J. industr. Med.*, **6**, 245.
DOLL, R., AND HILL, A. B.—(1950) *Brit. med. J.*, **2**, 739.
EARLE, W. R.—(1943) *J. nat. Cancer Inst.*, **4**, 165.
FALK, H. L., STEINER, P. E., GOLDFEIN, S., BRESLOW, A., AND HYKES, R.—(1951) *Cancer Res.*, **11**, 318.
FLORY, C. M.—(1941) *Ibid.*, **1**, 262.
GODLEWICZ, M.—(1949) *Nature*, **164**, 1132.
GOULDEN, F., AND TIPLER, M. M.—(1949) *Brit. J. Cancer*, **3**, 157.
HATCH, T., AND HEMEON, W.—(1948) *J. industr. Hyg. Tox.*, **30**, 172.
HIEGER, I.—(1946) *Cancer Res.*, **6**, 657.
LANDAHL, H. D., AND HERRMANN, R. G.—(1948) *J. industr. Hyg. Tox.*, **30**, 181.
'Leicester, Atmospheric Pollution in.'—(1945). London (H.M. Stationery Office).
MAY, K. R.—(1945) *J. sci. Instrum.*, **22**, 187.
MILLER, J. A., AND BAUMANN, C. A.—(1943) *J. Amer. chem. Soc.*, **65**, 1540.
MULINOS, M. G., AND COCKRILL, J. R.—(1938) *Arch. int. Pharmacodyn.*, **58**, 200.
OWENS, J. S.—(1923) *Trans med. Soc., Lond.*, **45**, 79.
PASSEY, R. D.—(1922) *Brit. med. J.*, **2**, 1112.
SAMBURSKY, F., AND WOLFSOHN, G.—(1940) *Trans. Faraday Soc.*, **36**, 427.
SCHOENTAL, R., AND SCOTT, E. J. Y.—(1949) *J. chem. Soc.*, 1683.
SILVERMAN, L.—(1948) 'Industrial Air Sampling and Analysis.' Pittsburgh (Industrial Hygiene Foundation).
*Idem* AND VILES, F.—(1948) *J. industr. Hyg. Tox.*, **30**, 124.
SONKIN, L. S.—(1946) *Ibid.*, **28**, 269.
WARREN, F. L.—(1943) *Biochem. J.*, **37**, 338.
WEIGERT, F.—(1948) *Cancer Res.*, **8**, 169.
WEIL-MALHERBE, H.—(1944) *Ibid.*, **4**, 102.
WEISS, J.—(1948) *Nature*, **162**, 372.
WILSON, I., AND LAMER, V.—(1948) *J. industr. Hyg. Tox.*, **30**, 265.
WYNDER, E. L., AND GRAHAM, E. A.—(1950) *J. Amer. med. Ass.*, **143**, 329.